# Subjective and objective financial toxicity among colorectal cancer patients: a systematic review

**DOI:** 10.1186/s12885-023-11814-1

**Published:** 2024-01-05

**Authors:** Meram Azzani, Zahir Izuan Azhar, Aimi Nadira Mat Ruzlin, Chen Xin Wee, Ely Zarina Samsudin, Sabah Mohammed Al-Harazi, Sarah Noman

**Affiliations:** 1 Department of Public Health Medicine, Faculty of Medicine, Universiti Teknologi MARA, Selangor, Malaysia; 2 Department of Early Clinical Exposure and Professional Personal Development, Faculty of Medicine, MAHSA University, Selangor, Malaysia; 3 Department of Community Health, Faculty of Medicine and Health Sciences, Universiti Putra Malaysia, Serdang, 43400 Malaysia

**Keywords:** Direct medical cost, Direct non-medical cost, Indirect medical cost, Catastrophic health expenditure, Perceived financial hardship

## Abstract

**Background:**

Colorectal cancer (CRC) is the third most common cancer type worldwide. Colorectal cancer treatment costs vary between countries as it depends on policy factors such as treatment algorithms, availability of treatments and whether the treatment is government-funded. Hence, the objective of this systematic review is to determine the prevalence and measurements of financial toxicity (FT), including the cost of treatment, among colorectal cancer patients.

**Methods:**

Medline via PubMed platform, Science Direct, Scopus, and CINAHL databases were searched to find studies that examined CRC FT. There was no limit on the design or setting of the study.

**Results:**

Out of 819 papers identified through an online search, only 15 papers were included in this review. The majority (*n* = 12, 80%) were from high-income countries, and none from low-income countries. Few studies (*n* = 2) reported objective FT denoted by the prevalence of catastrophic health expenditure (CHE), 60% (9 out of 15) reported prevalence of subjective FT, which ranges from 7 to 80%, 40% (6 out of 15) included studies reported cost of CRC management– annual direct medical cost ranges from USD 2045 to 10,772 and indirect medical cost ranges from USD 551 to 795.

**Conclusions:**

There is a lack of consensus in defining and quantifying financial toxicity hindered the comparability of the results to yield the mean cost of managing CRC. Over and beyond that, information from some low-income countries is missing, limiting global representativeness.

**Supplementary Information:**

The online version contains supplementary material available at 10.1186/s12885-023-11814-1.

## Introduction

Colorectal cancer is the third most common cancer type worldwide; almost 2 million cases were detected in 2020. Colorectal cancer is also the second most common cause of cancer death worldwide, accounting for 1 million deaths per year [1]. Colorectal cancer treatment costs vary between countries as it depends on policy factors such as treatment algorithms, availability of treatments and whether the treatment is government-funded [2]. For underinsured patients, their out-of-pocket expenses will be higher [3]. A term that is associated with this situation is referred to as financial toxicity, which is the adverse impact of out-of-pocket healthcare costs suffered by the patients [4].

Financial toxicity can generally be divided into subjective financial distress and objective financial burden. Subjective financial distress occurs as a result of increasing cancer-related expenditures and financial difficulties, on top of the anxiety and discomfort experienced by the patient over their disease. The objective financial burden is due to the direct expenses of the cancer treatment, which will increase progressively from the first time the patient is diagnosed. As the patient spends more on cancer treatment, his income and assets will decrease over time. This financial burden is relative to the income and assets of the household of the patient with cancer, which decreases over time [5].

Costs of management for colorectal cancer patients include costs for surgery, chemotherapy, radiotherapy and palliative care. For example, the mean cost for each patient and treatment going for surgery in China range between $5,301 - $5,489 [6]. Another study in Spain revealed the cost of surgery for patients with Stage 1 to Stage 4 colorectal cancer range between $11,373 - $14,236 [7]. However, for both China and Spain, universal health care has been implemented which has benefited the population.

Studies have looked into the financial toxicity among different types of cancer patients which include prostate cancer [8, 9], breast cancer [10, 11] and lung cancer [12, 13]. However, there is a lack of studies that focus on colorectal cancer patients. It is important to determine the extent of financial toxicity among these types of patients.

Therefore, the objective of this systematic review is to determine the prevalence and measurements of financial toxicity, including the cost of treatment, among colorectal cancer patients.

## Search strategy

### Methods

The systematic review was conducted according to the Preferred Reporting Items for Systematic Reviews and Meta-Analyses statement (PRISMA) [14] (supplementary file 1-PRISMA checklist). The registration number of the protocol is CRD42023399186.

#### Literature search strategies and study selection

The following literature databases were searched in January 2023: PubMed, Science Direct, Scopus and CINAHL databases were searched to find papers that reported FT. The primary outcome was to find the prevalence of objective and subjective FT due to CRC cancer management. The search was built on the following keywords and Medical Subject Headings which were based on the research question: population (patients), exposure (colorectal cancer), and outcome (financial toxicity/hardship/burden/stress) and their synonyms (Supplementary file 2: search strategy). Additionally, a manual search through the reference list of the eligible studies was applied. We included original quantitative research that reports the FT of CRC cost of treatment published before Jan. 2023. Papers of mixed cancer patients that reported the cost of each cancer separately were included if they involved the CRC cost. There was no limit on the design or setting of the study to minimize underreporting bias. In addition, studies that reported any cost of CRC, including direct medical, direct non-medical, and indirect costs were included. Medical research studies which include economic evaluation studies, reviews, qualitative studies, case series and case studies were excluded. This study did not assess the intangible cost as it is difficult to calculate their monetary value.

#### Data extraction and quality assessment

Selection and screening of titles, abstracts and full text was conducted independently by two authors with disagreements resolved via consensus or the involvement of a third author. One author performed the data extraction, and the second author checked them for completeness and accuracy. Key information extracted included the author, publication year, study type, research methods, study setting, main findings, and conclusion. The data was presented based on author date, type of cancer, study participants (sample size, socio-demographic characteristics such as age and gender), the prevalence of FT (subjective and objective), cost of illness, tools used to measure the FT, and quality scoring (Supplementary file 3). The prevalence of subjective FT is assessed using a questionnaire aimed at understanding the financial challenges individuals face due to healthcare expenses. The results were presented using a numerical description which is the proportion. However, the objective FT is measured using the prevalence of catastrophic health expenditure (CHE), which was defined as a healthcare cost-to-income ratio of more than 40% in the included studies.

The quality of all included papers was assessed using the Newcastle *-* Ottawa quality assessment scale (NOS) for longitudinal, cohort and cross-sectional studies (adapted for cross-sectional studies), which comprises three dimensions: selection, comfortability, and outcome. Each study was evaluated based on the NOS scale for fulfilling the established criteria in NOS for the 3 dimensions. An overall quality score was calculated by adding the number of stars for each category for a maximum total of 9. High-quality studies were defined as those with a score of 5 or higher, with higher scores suggesting a decreased likelihood of bias and higher quality [15] Disagreements between the two reviewers during full-text screening were reconciled via consensus or by the decision involving a third independent reviewer. The quality score can be found in Supplementary File 3.

## Results

### Description of studies

**Fig. 1:**
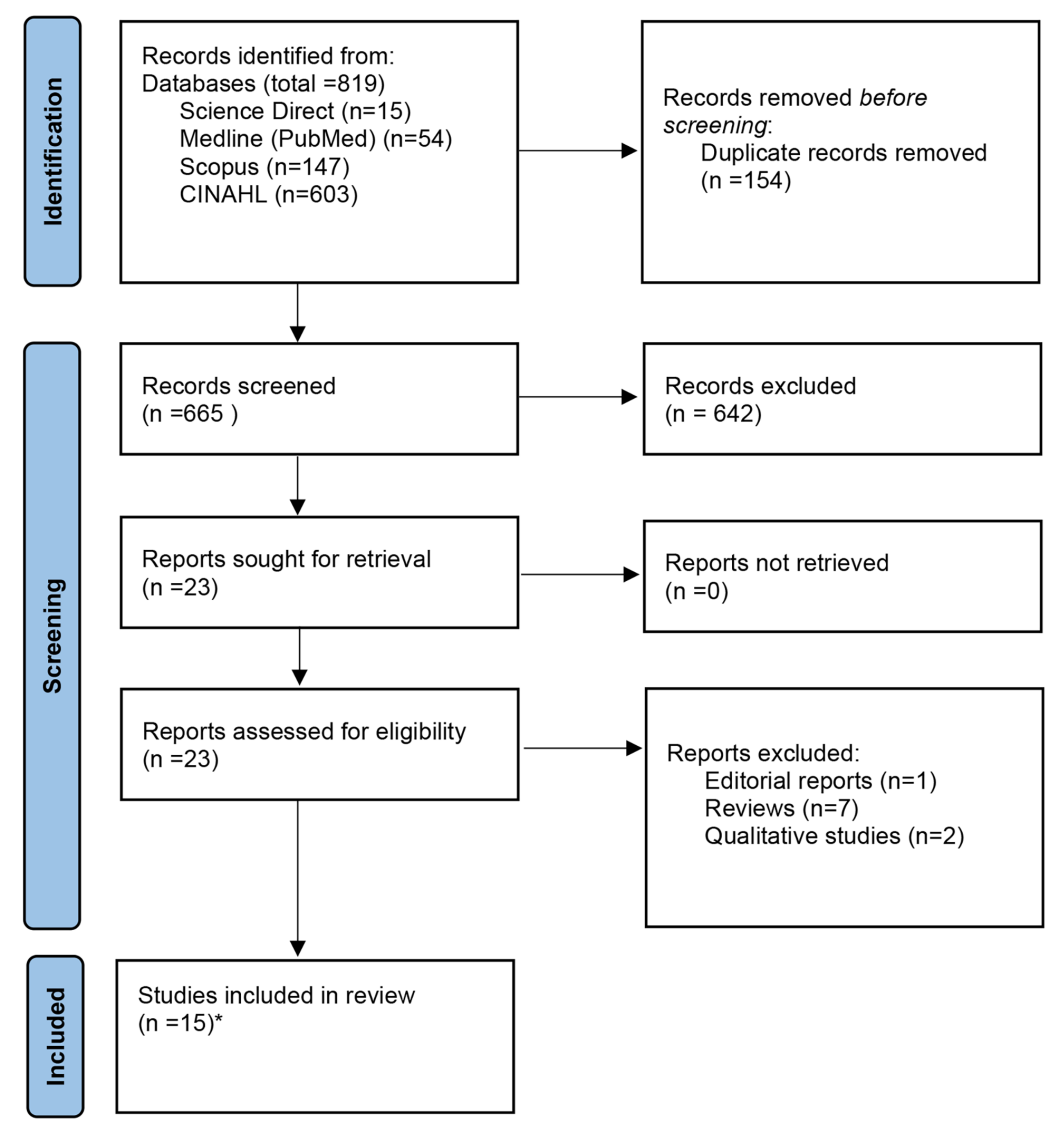
PRISMA flowchart *Two records were obtained by screening the citations of included studies

All search results were transferred to the Endnote X9 (Clarivate Analytics) which was used to manage the articles and manual searches. A total of 819 papers were identified through an online search and 2 papers through a manual search. Then, the duplicate papers were eliminated (*n* = 59). The titles and abstracts of the remaining 665 papers were screened and selected independently by two reviewers according to the established inclusion and exclusion criteria. Subsequently, a total of 23 papers were retained for full-text review. After a full-text review of the 23 papers, 15 were selected. The eligibility of included papers was agreed upon by all authors. The PRISMA flow chart demonstrated the screening process (Fig. 1) [16]. The studies in this review were conducted globally including Malaysia (*n* = 2) [17, 18], USA (*n* = 5) [19–23], Australia (*n* = 1) [24], China (*n* = 3) [25–27], Ireland (*n* = 3) [28–30] and Iran (*n* = 1) [31]. A total of 246,915 colorectal cancer patients took part in 15 studies worldwide, with samples ranging from 104 to 237,754 patients. Most studies included participants with any stage (I-IV) of cancer; however, one research included patients with stage III cancer [20] and another study included patients with stage IV cancer [21].

### Measurement of objective financial toxicity

Included studies rarely focused specifically on quantifiable indicators of FT. Only 2 studies measured the objective FT in terms of the prevalence of catastrophic health expenditure (CHE), which was defined as a healthcare cost-to-income ratio of more than 40% in the included studies [18, 31] and found it to be 68.5% in Iran [30] and 47.8% in Malaysia [18].

### Measurement of subjective financial toxicity

Out of the 15 research that were included in this review, 9 studies [17, 19–21, 24, 27–29] provided data on subjective FT. There was a wide variation in the measures of FT among the studies. Two of them employed the COmprehensive Score for Financial Toxicity (COST), which consists of 11 items and has a score range of 0–44 [19, 27]. Lower COST values denote greater FT. Patients with FT were categorised by Mo et al. using the median COST score, with individuals scoring fewer than 21 being classified as having experienced FT [26]. Additionally, four studies that employed a 4–7 point Likert scale to evaluate the prevalence of subjective FT reported prevalence was between 20.9% and 41% [17, 20, 25, 26, 29]. Moreover, one study utilised four questions with “yes” or “no” responses to gauge subjective FT; those who gave “yes” replies to at least one of the four questions were deemed to be suffering from financial toxicity [21]. Gordon et al. used three 3 domains to measure the FT which are: perceived prosperity, financial strain and ability to raise money ($2000) [24]. According to Edward et al. 2021, the FT included both material and psychological difficulty; the material FT was estimated to be 80% and the psychological FT was calculated using COST [19]. The details of the prevalence of subjective FT and the tools used to measure it can be found in Table 1.

**Table 1:** The prevalence of subjective financial toxicity in included studies

Author, Year	Country	Cancer stage^a^	Year of Research	Sample size	Prevalence of FT^b^ (%)	Tools used
Azzani et al. 2016 [17]	Malaysia	Stage I = VI	2016	138	20.9%	5-point Likert scale (‘very difficult’, ‘difficult’, ‘somewhat difficult’, ‘not that difficult’ or ‘not difficult at all’). A categorization was achieved according to the reporting of a difficult/very difficult (yes/no).
Sharp et al. 2018 [29]	Europe	Stage I = VI	2007–2009	493	41% had financial stress, 39% financial strain, 32% reported both financial stress and financial strain	7 Likert scale more diff more concern to much less concern, collapse into more diff/concern, no change, less diff/concern financial stress was assessed as the impact of the cancer diagnosis on the household’s ability to make ends meet, financial strain as the impact on the individual (i.e. how the respondent had felt about their household’s financial situation since their cancer diagnosis).
Edward et al. 2021 [19]	USA	Stage I = VI	2019	104	80% had material burden and the mean COST was 24.5	A material burden: total score was formed by summing seven indicator variables bankruptcy, borrowing money or going into debt, making financial sacrifices, being worried about having to pay large medical bills, being unable to cover treatment costs, and not being able to receive care because of costs. Psychological aspects: using the COST (COmprehensive Score for Financial Toxicity) questionnaire
Gordon et al. 2017 [24]	Australia	Stage I = VI	Jan 2010 to Sep. 2011	187	1–0.6% answered as poor for 1st domain, financial strain reported by 15% and 7%, difficult to raise money 41% and 33% at 6 and 12 months, respectively	FT questionnaire of 3 domains: perceived prosperity (prosperous, very comfortable, reasonably comfortable, just getting along, or poor or very poor), financial strain (couldn’t pay utilities on time, couldn’t pay my mortgage or rent on time, sold something, went without a meal, unable to heat home, ask for financial help from friends or family, asked to financial help form organization) ability to raise money ($2000) (I could easily raise money, unable/difficult to raise money)
Shankaran et al. 2012 [21]	USA	Stage III colon cancer	2008–2010	284	71.3%	Major financial hardship (MFH) was defined as 1 or more of the following during the 12 months following enrolment: accumulating a debt of any amount, selling a home, refinancing a home, experiencing a higher than 20% income decline, or borrowing money from family and/or friends.
Hanly et al. 2018 [28]	Ireland	Stage I-VI	2010	496	40.9% experienced objective stress and 39.4% experienced subjective strain.	7 Likert scale more diff more concern to much less concern, collapse into more diff/concern, no change, less diff/concern financial stress was assessed as the impact of the cancer diagnosis on the household’s ability to make ends meet, financial strain as the impact on the individual (i.e. how the respondent had felt about their household’s financial situation since their cancer diagnosis).
Mo, M et al. 2023 [27]	China	Stage I = VI	2022	250	Median = 21, 52.8% had FT	COST (COmprehensive Score for financial Toxicity) questionnaire
Regenbogen, Set al. 2014 [20]	USA	Stage III	2011–2013	937	No sum of score, the results compared the FT between those with complications and without it using the 7 items of FT and 2 items on worry and composite financial burden	The personal financial burden was evaluated using a seven-item checklist: “I had to use savings,” “I had to borrow money or take out a loan,” “I could not make payments on credit cards or other bills,” “I cut down on spending for food and/or clothes,” “I cut down on spending for health care for other family members,” “I cut down on recreational activities,” and “I cut down on expenses in general.” with higher scores denoting increased financial burden. The composite measure was: (“My illness has had no impact on my finances”) and a single question about financial worry (“How much do you worry about financial problems that have resulted from your colorectal cancer and its treatment?” Worry was evaluated on a 5-point Likert scale that we dichotomized by our previous work (in which scores of 1–3 were considered low, and scores of 4–5 were considered high)
Huang et al. 2017 [25]	China	all stages	September 2012 to December 2014	2356	75.0% of the families perceive an unmanageable burden (47.4% heavy, 27.6% overwhelmed); only 18.3% perceived a somewhat manageable burden, and 6.7% perceived no burden at all. The	Questions were asked, “Which of the following accurately describes your family’s financial pressure from your disease?” and offered four response options: “not at all,” “somewhat but manageable,” “heavy,” and “overwhelmed.” We classified “not at all” and “somewhat but manageable” as manageable burdens; we classify

^a^Cancer stage: Unless indicated otherwise, cancer stage indicates colorectal cancer stage; ^b^Prevalence of FT: Prevalence of financial toxicity; USA: United States of America; COST: COmprehensive Score for Financial Toxicity

#### Cost of cancer management

The cost of cancer management included direct medical costs, direct non-medical cost and indirect costs. It was reported in six of the included studies [17, 18, 23, 25, 26, 30] which are from China, USA, Malaysia and Ireland. The included studies were published between 1999 and 2017. All studies considered the four cancer stages except Li et al. 2016 where he considered the total cost of CRC treatment regardless of disease stage (Table 2).

### Direct medical costs

Data on mean direct medical costs from different perspectives were reported in five studies in total [17, 18, 23, 25, 26, 30]. Among studies that were included, the period during which the expenditures were incurred varied widely, including annual cost [17, 18, 25, 26], colorectal cancer survival [30] and 4 years cost [23]. In regards to cost perspective, the patient perspective was employed in three research [17, 18, 30], one study considered the national health care perspective [23] and one study reported the cost from the patient perspective to calculate the non-medical cost and from the insurance plan database to find out the medical cost [25]. Detailed cost data can be found in Table 2.

### Direct non-medical costs

The direct non-medical cost was included in only two studies [25, 30] where the cost in Europe was found to be €510 (USD703.8) [29] and 5588 CNY (USD901.2) in China [25] (Table 2).

### Indirect cost

Two studies in total reported the indirect cost of colorectal cancer management [18, 25] where it was USD452.2 in Malaysia [18] and 6652CNY (USD1,072.9) in China [25] (Table 2).

**Table 2:** Direct medical, non-medical and indirect costs in included studies

Author, Year	Country	Stage of Cancer^a^	Year of Research	No. of patients^b^	Direct medical cost	Direct non-medical	indirect cost	Perspective
Azzani et al. 2016 Azzani et al. 2017 [17, 18]	Malaysia	all stages	2013	138	RM 6544.5 (USD 2045.1) for stage I, RM 7790.1 (USD 2434.4) for stage II, RM 8799.1 (USD 2749.7) for stage III and RM 8638.2 (USD 2699.4) for stage IV	NA	USD 452.2	Patient
O Céilleachair et al. 2017 [30]	Ireland	all stages	October 2007–September 2009	497	Average OOP: €1589 (USD 2192.8) among colorectal cancer survivors	€510(USD703.8)	NA	Patient
Seifeldin & Hantsch 1999 [23]	USA	all stages	1991–1994	The mean number of admissions: 237,754 per year	Total hospital charge is USD 4.5 billion per year (4 years period)	NA	NA	National HC
Huang et al. 2017 [25]	China	all stages	September 2012 to December 2014	2356	51,366 CNY (USD 8,284.8) for stage I to 75,673 CNY (USD 12,205.3) for stage IV disease	5588 CNY(USD 901.2) per CRC patient	mean wage loss amounted to 6652 CNY (USD 1,072.9).	Patient and health insurance
Li et al. 2016 [26]	China	Not mentioned	January to December 2013	1211	USD 757,432.26	NA	NA	new Rural Cooperative Medical Scheme (NCMS)

^a^Stage of Cancer: Unless indicated otherwise, stage of cancer indicates stage of colorectal cancer; ^b^No. of patients: number of patients; RM: Malaysian Ringgit; USD: United States Dollar; NA: not available; National HC: National health care; $: dollar sign; OOP: out-of-pocket; €: euro sign; USA: United States of America; CNY: Chinese yuan renminbi; CRC: colorectal cancer; NCMS: new rural cooperative medical scheme

## Discussion

The present review examined the prevalence and measurement of FT among CRC patients. Fifteen studies were included; the majority (*n* = 12, 80%) were from high-income countries, others were from middle-income countries, and none from low-income countries. They were published between 1999 and 2023. Several main findings were identified, they were (i) few studies (*n* = 2) reported objective FT denoted by the prevalence of CHE, (ii) 60% (9 out of 15) reported a prevalence of subjective FT, which ranges from 7 to 80%, (iii) large variation and lacking standardized measurement tool to quantify subjective FT, (iv) 40% (6 out of 15) included studies reported cost of CRC management– annual direct medical cost ranges from USD 2045 to 10,772 and indirect medical cost ranges from USD 551 to 795.

While two studies regarded the objective FT as the prevalence of CHE, nine studies referred to subjective FT using different measurement scales. Five studies applied scaled questions (e.g., Likert-scale), two used the COST instrument, one used dichotomous questions, and another applied multidimensional dichotomous questions. Unstandardized measurement tools on subjective financial distress hindered the comparison of results between regions globally or countries by income classification, making it more challenging for global health players and state health authorities to plan an appropriate resource distribution in cancer management [32]. Future research shall consider developing a standardized instrument to measure FT using six domains– active financial spending, use of passive financial resources, psychosocial responses, support seeking, coping with care, or coping with one’s lifestyle [33].

Fifteen studies included in this review reported data from six countries that have a life expectancy of more than 75 years, and most were from high-income countries. Cancer patients living in countries with advanced medical modalities will have better survivorship provided that they have the financial assistance to receive the treatment and enough savings/assistance to support the incomeless days. Throughout the synthesis, we discovered that US data showed a high prevalence of subjective FT at more than 70%, despite having a similar proportion (17%) of people living below 50% of median income compared to Malaysia [34]. This might be because Malaysia, which has a dual-tier healthcare system, offers more affordable options in the public sector as the cost of care is heavily subsidized by the government. In contrast to the USA, where there is a reliance on private health insurance, where access to comprehensive coverage is contingent upon factors like employment status. Consequently, this review highlights the point of focusing on the financial burden attributed to CRC in unemployed ageing society to ensure healthy aging and good quality of life is one of the major determinants of healthy living among the elderly.

A paucity of studies reporting the direct non-medical [25, 30] and indirect cost [17, 18, 25], which could directly contribute to the subjective FT. The responsibility of clinicians in providing high-quality treatment is typical, however, their role in assisting to leverage financial burden and distress to the patients in the short and long term might be emphasized and supported by the national health insurers or social welfare department based on the healthcare financing system in the country [6]. Without a proper and comprehensive system to acknowledge and quantify the cost incurred to the patients, family, community and the nation, it becomes difficult to engage key stakeholders in paying attention to the public health insurance system and subsequently implement proper policy to incentivize cancer survivors [32]. Therefore, it is recommended for future researchers to obtain a situational analysis of financial burden regarding direct non-medical and indirect costs, particularly in low-income countries.

This is the first review analyzing the FT among CRC patients and the cost of CRC management. Most of the included studies recruited CRC patients from all stages, making the study population homogenous. However, a systematic review naturally presents publication bias; however, authors have attempted to minimize it by obtaining data from all available sources from the electronic databases, citations, and manual search. Secondly, a lack of consensus in defining and quantifying financial toxicity hindered the comparability of the results to yield the mean cost of managing CRC. Over and beyond that, information from some low-income countries is missing, limiting global representativeness.

## Conclusion

Most of the studies included were conducted in high-income countries, with none originating from low-income nations. FT is prevalent and has emerged as a significant concern, even within publicly funded health systems with universal coverage. There is a need for additional research, particularly from low-income countries, to investigate the financial toxicity of CRC. Furthermore, it is essential to develop and validate a tool for quantifying FT in CRC patients through further research.

### Electronic supplementary material

Below is the link to the electronic supplementary material.

Supplementary Material 1

Supplementary Material 2

Supplementary Material 3

## Data Availability

All data generated or analysed during this study are included in this published article.
