# Supplementary material for: Subjective and objective financial toxicity among colorectal cancer patients: a systematic review
Source: BMC Cancer. 2024 Jan 5;24:40. doi: 10.1186/s12885-023-11814-1 (PMC10770883; doi:10.1186/s12885-023-11814-1)
Supplement: Supplementary file 2 — Supplementary Material 2 [file 12885_2023_11814_MOESM2_ESM.doc]

Database: Medline via PubMed platform

Number of hits: 54

Date: 25.1.2023

Seach keywords

(((((("financial burden"[Title/Abstract]) OR ("financial toxicity"[Title/Abstract])) OR ("financial hardship"[Title/Abstract])) OR ("financial stress"[Title/Abstract])) OR ("catastrophic health expenditure")) AND ("colorectal cancer"[Title/Abstract])) AND (Patients[Title/Abstract])

("financial burden"[Title/Abstract] OR "financial toxicity"[Title/Abstract] OR "financial hardship"[Title/Abstract] OR "financial stress"[Title/Abstract] OR "catastrophic health expenditure"[All Fields]) AND "colorectal cancer"[Title/Abstract] AND "Patients"[Title/Abstract]
