# Supplementary material for: Subjective and objective financial toxicity among colorectal cancer patients: a systematic review
Source: BMC Cancer. 2024 Jan 5;24:40. doi: 10.1186/s12885-023-11814-1 (PMC10770883; doi:10.1186/s12885-023-11814-1)
Supplement: Supplementary file 3 — Supplementary Material 3 [file 12885_2023_11814_MOESM3_ESM.doc]

Table S2: Data extraction table

| **NO.** | **Author-year** | **Country and setting** | **Study design-sampling** | **Cancer Type and stage** | **Tools to measure financial toxicity** | **results** | **conclusion** | **scoring** |
| --- | --- | --- | --- | --- | --- | --- | --- | --- |
| 1. | Azzani et al. 2016 | Malaysia,  over the course of 1 year (2013). | Study design: a prospective, longitudinal study  Sample size: 138 | Colorectal cancer - stage (I–IV). | Five-point Likert scale (‘very difficult’, ‘difficult’, ‘somewhat difficult’, ‘not that difficult’ or ‘not difficult at all’). | At three time intervals, the prevalence of perceived financial difficulty among colorectal cancer patients in the first year after diagnosis was highest for Somewhat difficult: 71.7% during the diagnostic phase, 47.9% after six months, and 35.3% after the last six months.  The total one-year patient cost (both direct and indirect) increased with stage of colorectal cancer: -RM 6544.5 (USD 2045.1) for stage I  -RM 7790.1 (USD 2434.4) for stage II  -RM 8799.1 (USD 2749.7) for stage III  - RM 8638.2 (USD 2699.4) for stage IV. | The majority of CRC patients and their families incurred high costs for CRC treatment and perceived healthcare payment as difficult, especially during the advanced stages of cancer. | 6 |
| 2. | Azzani et al. 2017 | Malaysia,  over the course of 1 year (2013). | Study design: a prospective, longitudinal study.  Sample size:138 | Colorectal cancer - stage (I–IV). | CHE defined healthcare cost-to-income ratio of more than 40% | The prevalence of CHE was calculated to be 47.8% in the first year after diagnosis.  The mean total cost of CRC treatment after one year was RM8306.9 (US$2.595.9).  The highest cost was noticed in the late stages (III and IV)  Stage I: Total cost: 6544.5 (US$2045.1))  Stage 2 (Total cost: 7790.1 (US$2434.4))  Stage 3 (Total cost: 8799.1 (US$2749.7))  Stage 4 (Total cost: 8638.2 (US$2699.4)) | In this study, approximately half of CRC patients and their families experienced CHE due to healthcare payments. | 6 |
| 3. | Edward et al. 2021 | USA, January to November 2019 | Study design: cross-sectional.  Sample size: 104 | Colorectal cancer - stage (I–IV) | Cancer survivors' financial hardship was measured by objective (material) and subjective (psychological) measures. A material burden total score was determined by combining seven indicator variables from the Medical Expenditure Panel Survey - Experiences with Cancer Survivorship Supplement.  Psychological aspects of financial hardship, known as financial toxicity (FT), were measured using the 11-item Comprehensive Score for Financial Toxicity (COST) tool (scored from 0 to 44, with higher scores indicating worse FT). | 80% of participants reported experiencing one or more material burdens due to their cancer.  The mean financial toxicity score was 24.5 (10.4); ranging from 3 to 43. | The findings suggest that programmes and interventions aimed at improving health insurance literacy and numeracy are required to reduce financial toxicity and hardships among colorectal cancer survivors. | 8 |
| 4. | Gordon et al.2017 | Australia, January 2010-September 2011. | Study design: prospective, population-based study enrolling 45–64-year-better understand old men and women with colorectal cancer.  Sample size: 187 | Colorectal cancer:  Early stage  :- 71 (65%)  - Late 38 (35%) | The financial hardship was evaluated by three questions based on the Household Income and Labour Dynamics in Australia (HILDA) survey. 1. Perceived prosperity  2.Financial strain was  3.Ability to raise money | A higher proportion of workers with colorectal cancer reported financial strain (money shortage for living essentials) at 6 months (15%) but this decreased and was comparable to the comparison group at 12 months (7%). | Cancer support services should help health professionals and patients identify and alleviate financial burdens. | 6 |
| 5. | Hanly et al. 2018 | Ireland  April and September 2010” | Survey,  sample size: 496 | Colorectal cancer stage (I–IV) | Objective stress was measured by asking respondents “has your cancer diagnosis made your household‟s ability to make ends meet?” and offering seven possible ranging from “much more difficult” to “much less difficult”.  The subjective strain was assessed by asking, “since your cancer diagnosis, how have you felt about your household‟s financial situation?” and offering seven possible responses ranging from "much more concerned" to "much less concerned". | Approximately 40.9% of survivors or 39.4% experienced objective stress or subjective strain. | Cancer-related financial objective stress and subjective strain are common in colorectal cancer survivors, even in countries where all citizens are entitled to publicly-funded care, but financial coping strategies differed significantly.  These findings will help inform targeted measures to alleviate financial hardship across disparate health care systems and survivor groups. | 8 |
| 6. | Huang et al. 2017 | China, September 2012 to December 2014 | Multicenter, hospital-based, cross-sectional stud.  Sample size: 2356 | Colorectal cancer stage (I–IV) | Expenditure data for the entire course of illness up to the survey date by clinical visit—both outpatient and inpatient, occurring both within and outside the surveyed hospitals —i.e., the start date of treatment, the length of hospitalization, the total medical expenditure, the total and detailed non-medical expenditure | The overall average direct expenditure per patient was estimated to be 67,408 CNY, with expenditures for stages I, II, III, and IV disease being 56,099 CNY, 59,952 CNY, 67,292 CNY, and 82,729 CNY, respectively.  Non-medical spending accounted for 8.3% of total spending.  A newly diagnosed patient's one-year out-of-pocket expenditure was 32,649 CNY, accounting for 59.9% of their previous-year household income and causing 75.0% of families to face an unmanageable financial burden. | Direct expenditure for CRC diagnosis and treatment appeared catastrophic for patients in China, and non-medical expenditure was unavoidable. | 7 |
| 7. | Mo et al. (2023) | China, January 2022 to June 2022 | Study design:  single-institution, cross-sectional pilot study.  Sample size: 250 | Non-metastatic colorectal cancer stage (I–IV) | The 11-item Comprehensive Score for Financial Toxicity (COST) questionnaire was used to assess financial toxicity. It was scored from 0 to 44, with each item being rated on a 4-point scale. Low values represented financial toxicity from more significant treatment modalities. | 52.8% of colorectal cancer survivors reported financial toxicity. | Financial toxicity is common among survivors of non-metastatic colorectal cancer. Financial toxicity was linked to young age, lower annual household income, unemployment, chemotherapy, and a lack of social support. | 9 |
| 8. | Regenbogen al. (2014) | USA, August 2011 -March 2013. | Survey,   Sample size: 937 | colon or rectal cancer, stage III | The personal financial burden was evaluated using a seven-item checklist (obtained from the national Consumer Bankruptcy Project) that asked participants to indicate whether or not they had experienced any of the following: “I had to use savings,” “I had to borrow money or take out a loan,” “I could not make payments on credit cards or other bills,” “I cut down on spending for food and/or clothes,” “I cut down on spending for health care for other family members,” “I cut down on recreational activities,” and “I cut down on expenses in general.” The patients also were asked how much cumulative time they missed work as a consequence of their cancer and its treatment. | Those patients with complications after surgery (24%) had significantly higher composite financial burden (P < .001 for trend): they were more likely to spend savings (40% vs 31%; P = .01), borrow or take loans (18% vs 11%; P = .007), fail to make credit card payments (18% vs 11%; P = .005), reduce spending for food or clothes (38% vs 27%; P = .001), and decrease recreational activities (41% vs 33%; P = .03). They took significantly longer to return to work (P =.009) and were more likely to have significant financial anxiety (61% vs 52%; P =.01). | Complications from CRC surgery have significant personal financial consequences as well as morbidity. Financial stress reduces the quality of life and may prevent adherence to recommended treatments. As a result, patients who develop complications may require not only additional clinical care but also financial assistance and services. |  |
| 9. | Shankaran et al. 2022. | USA. June 2016 to January 2019 | A prospective cohort study. Sample size: 320 | Metastatic colorectal cancer (mCRC) patients  aged 18 years or older and newly diagnosed with stage IV colorectal cancer within 120 days of registration | A 20-item questionnaire administered after consent (baseline survey) and a 27-item follow-up questionnaire administered at scheduled clinical visits (3, 6, 9, and 12 months) after registration were used to assess major financial hardship (MFH).  These financial questionnaires were adapted from a questionnaire administered to a population-based sample of stage III colon cancer patients in the Seattle-Puget Sound region, the Medical Expenditures Panel Survey | The cumulative incidence of major financial hardship (MFH) at 12 months was 71.3% (95% confidence interval 14 65.7% to 76.1%). | Despite having access to health insurance, nearly three out of every four mCRC patients experienced MFH.  These findings highlight the importance of clinic and policy solutions that shield cancer patients from financial harm. | 6 |
| 10. | Sharp et al. 2018 | Ireland, January 2010 | Cross-sectional postal survey.  Sample size: 493 | Colorectal cancer survivors diagnosed 6 to 37 months prior, stage (I–IV) | The questionnaire included one objective and one subjective measure of cancer-related financial impact: cancer-related financial stress and cancer-related financial strain. Cancer-related financial stress was defined as the impact of the cancer diagnosis on the ability of the household to make ends meet, whereas cancer-related financial strain was defined as the impact on the individual (ie, how the respondent had felt about the financial situation of their household since their cancer diagnosis). | Overall, 41% reported cancer-related financial stress, 39% financial strain, and 32% both financial stress and financial strain.  Those who reported cancer-related financial stress postdiagnosis had a significantly higher risk of low health-related quality of life than those who reported no change in financial stress postcancer (OR = 2.54 (95% CI, 1.62-3.99)). The odds of having low health-related quality of life after diagnosis were also significantly higher (OR =1.73 (95% CI, 1.09-2.72)). The odds ratio for those who experienced both cancer-related financial stress and financial strain was 2.59 (95% CI, 1.59-4.22). | Four out of every ten colorectal cancer survivors reported a financial impact from the disease. Cancer-related financial stress and strain were found to be significantly related to poor health-related quality of life. Additional research is needed to better understand how objective and subjective financial distress influence survivors' health-related quality of life in order to inform support strategies. | 8 |
| 11. | Veenstra et al. 2014. | USA,  August 2011 -March 2013 | Study type: survey  Sample size: 956 | Colon or rectal cancer: Stage III | This study aims to generate and evaluate a novel patient-reported indicator of personal financial hardship during CRC treatment.  7 binary questions were used to evaluate the patients' personal financial burden by asking them how the CRC or its treatment affected their finances. These measures were adapted from those recommended by the National Consumer Bankruptcy Project.  The questions about the financial burden were:  (1) I had to use savings.  (2) I had to borrow money or take out a loan.  (3) I could not make payments on credit cards or other bills.  (4) I cut down on spending for food and/or clothes.  (5) I cut down on spending for health care for other family members.  (6) I cut down on recreational activities.  (7) I cut down on expenses in general.  This composite 6-item measure has a score range of 0–6 (higher scores denote increased financial burden).  The questions about the patient’s Worry include “  How much do you worry about financial problems that have resulted from your colorectal cancer and its treatment?”  worry was categorized as low (1–3) or high (4–5). | 6 items had factor loadings that were >0.4 and were therefore included in the composite score. (Cronbach's α = 0.79) There was high internal consistency.  The mean financial burden score was 1.72 (SD = 1.83). The scale ran from 0 to 6, with 366 (38%) respondents supporting no financial burden measures. A total of 277 (29%) reported 1-2 measures, 223 (23%) reported 3-4, and 90 (9%) reported 5.  Financial burden scores were considerably higher for the 812 (85%) who reported using chemotherapy than for those who did not (mean burden score: 1.88 vs. 0.88, P 0.001).  The commonly endorsed measure was “I cut down on expenses in general,” (48%). The least frequently endorsed was “I cut down on spending for health care for other family members” (5%). Inter-item correlations ranged from 0.14 to 0.61.  30% of those polled agreed with the statement "My illness has had no effect on my finances." 94% of those surveyed did not support any financial burden items (composite burden score = 0). Similarly, 54% of 562 respondents (60%) with low levels of financial worry had a composite financial burden score of 0.  Worry and financial burden were closely related in general: 70% of respondents had concordant worry and burden scores (Pearson Correlation Coefficient = 0.625, P 0.001). 4% had low worry but a high burden score (3-6), while 26% had high worry but a low burden score (0–2). | This study created a composite measure of personal financial burden among a population-based sample of patients with stage III CRC. The study encourages the use of this measure in identifying patients at risk of increased burden.  The younger working poor and those who had used chemotherapy endorsed the greatest burden. These vulnerable patient groups may benefit from policy interventions that provide economic support as they undergo potentially life-saving cancer treatment. | 5 |
| 12. | Li et al. 2016 | China, January to December 2013 | The study obtained claim data for three gastrointestinal cancers - esophageal, gastric, and colorectal - from Hua County's new Rural Cooperative Medical Scheme (NCMS) Management System.  Sample size: 1211 | Esophageal cancer, gastric cancer (includes cardia cancer and non-cardia gastric cancer), and colorectal cancer  No information about the colorectal cancer stages | The study is mainly to evaluate the financial burden of gastrointestinal cancer.  Cancer-specific health economic indicators were calculated to assess the financial burden under the local NCMS's protection. | The total cost of esophageal cancer was 2.7–3.6 times higher than that of gastric cancer and colorectal cancer, respectively, because of the high incidence of oesophageal cancer.  Colorectal cancer: $757 432.26 | Meanwhile, county-level health officials should be trained to increase their management capacity so that dynamic regulation of local NCMS reimbursement policy can take into account the pattern of local disease burden. | 8 |
| 13. | Piroozi et al. 2018. | Iran, December 2017 to August 2018. | A cross-sectional study.  Sample size: 189 | Gastrointestinal cancer:  Esophageal cancer  (22.4%)  Stomach cancer  (21.1%)  Rectum cancer (8.1%)  Colorectal cancer (33.5%)  Others (14.9%) | Measure the catastrophic health expenditures (CHE) among households with gastrointestinal cancer patients.  A World Health Survey questionnaire was used to collect data. A method developed by the World Health Organization with a threshold of 40% of a household's capacity to pay was used to calculate the proportion of households facing CHE. | 68.5% (37/54) of colorectal cancer households experienced CHE, | The proportion of households affected by CHE was very high. This may indicate a weakness in the health system as well as health insurance, or a weakness in the Health Transformation Plan (HTP) in providing financial protection to vulnerable populations. | 9 |
| 14. | Seifeldin & Hantsch et al. 1999 | United States  The HCUP includes up to 15 diagnosis  codes from the International Classcjkation  of Diseases, Ninth Revision, Clinical Mod-  ification (ZCD-9-CM) | N=237,754  Population aged 50 years ~60 years. | Colon cancer | To estimate the economic burden of hospitalizations for colon cancer.  To assess the relationship  between risk factors, including age, and  treatment charges and to estimate the  number of hospital admissions for colon cancer through the year 2050. | At -$20,000 per admission, charges averaged >$4.5 billion per year during the 4 years studied. Total annual admission charges increased from $3.90 billion to $5.14 billion over the 4-year period | Interventions to decrease the incidence and mortality of colon cancer are needed and should ultimately reduce associated costs. Until such interventions are developed, the economic burden of colon cancer will continue to increase as the population ages | 5 |
| 15. | O Céilleachair et al. 2017 | Ireland, National Cancer Registry | All cases of primary, invasive colorectal cancer in Ireland diagnosed October 2007–September  2009,  questionnaires were returned (response rate = 39%).  N=497 | Colorectal cancer, stage I, II, II, IV, and unknown. | Investigate out-of-pocket costs (OOPCs) incurred by colorectal (CRC) medications and indirect  costs). | The average OOPC was €1589. | Greater attention  should be paid for the development of services to help  survivors manage the financial and economic burden of  cancer. | 8 |
